# Examining a Resilience Mental Health App in Adolescents: Acceptability and Feasibility Study

**DOI:** 10.2196/38042

**Published:** 2023-03-22

**Authors:** Daniel K Elledge, Simon Craddock Lee, Sunita M Stewart, Radu Pop, Madhukar H Trivedi, Jennifer L Hughes

**Affiliations:** 1 Cook Children’s Medical Center Fort Worth, TX United States; 2 Department of Population and Data Sciences University of Texas Southwestern Medical Center Dallas, TX United States; 3 Department of Psychiatry University of Texas Southwestern Medical Center Dallas, TX United States; 4 Children's Medical Center Dallas, TX United States; 5 Center for Depression Research and Clinical Care Department of Psychiatry University of Texas Southwestern Medical Center Dallas, TX United States; 6 Peter O’Donnell Jr Brain Institute University of Texas Southwestern Medical Center Dallas, TX United States; 7 Nationwide Children's Hospital Columbus, OH United States; 8 Department of Psychiatry and Behavioral Health The Ohio State University Columbus, OH United States

**Keywords:** youth, prevention, resilience, mental health app, mobile phone

## Abstract

**Background:**

Resilience is defined as the ability to rely on internal characteristics and external strengths to adapt to adverse events. Although universal resilience-enhancing programs are effective for adolescents, there is a need for interventions that are more easily accessible and can be customized for individual teens. Phone apps are easy to use, can be tailored to individuals, and have demonstrated positive effects for mental health outcomes.

**Objective:**

This study aimed to examine the feasibility and acceptability of a resilience app for adolescents. This app aimed to enhance resilience through modules focused on depression prevention, stress management, and healthy lifestyle approaches containing videos, measures, and practice suggestions. Furthermore, the study aimed to evaluate the effect of short-term app use on changes in resilience.

**Methods:**

In study 1, individual interviews and focus groups were conducted with adolescents, parents, teachers, and clinicians to discuss possible incentives for using a mental health app, the benefits of app use, and concerns associated with app use. Feedback from study 1 led to ideas for the prototype. In study 2, individual interviews and focus groups were conducted with adolescents, parents, teachers, and clinicians to gather feedback about the resilience app prototype. Feedback from study 2 led to changes in the prototype, although not all suggestions could be implemented. In study 3, 40 adolescents used the app for 30 days to determine feasibility and acceptability. Additionally, resilience and secondary mental health outcomes were measured before and after app use. Dependent samples 2-tailed *t* tests were conducted to determine whether there were changes in resilience and secondary mental health outcomes among the adolescents before and after app use.

**Results:**

Multiple themes were identified through study 1 individual interviews and focus groups, including app content, features, engagement, benefits, concerns, and improvement. Specifically, the adolescents provided helpful suggestions for making the prototype more appealing and functional for teen users. Study 2 adolescents and adults reported that the prototype was feasible and acceptable through the Computer System Usability Questionnaire (mean 6.30, SD 1.03) and Mobile App Rating Scale (mean 4.08, SD 0.61). In study 2, there were no significant differences in resilience and mental health outcomes after using the app for 30 days. There was variation between the participants in the extent to which they used the app, which may have led to variation in the results. The users appeared to prefer the depression module and survey sections, which provided mental health feedback.

**Conclusions:**

Qualitative and quantitative data provide evidence that youth are interested in a resilience mental health app and that the current prototype is feasible. Although there were no significant mental health changes in study 3 users, practical implications and future directions are discussed for mental health app research.

## Introduction

### Background

Adolescence is an important period for brain development, identity formation, and the development of autonomy. Approximately 10% to 20% of youth experience mental health problems, which can cause physical, emotional, or social impairment [1,2]. Adolescent mental health problems have worsened since the COVID-19 pandemic started in March 2020 [3]. Although many interventions focus on the reduction of psychological symptoms, only a few focus on the prevention of mental illness and bolstering resilience [4].

Werner and Smith [5] first used the term resilience to describe the ability of children who experienced poverty and domestic violence to *bounce back* and live stable lives as adults. Werner and Smith [5] found initial protective factors (eg, a stable adult role model), which helped increase youth’s resilience in the presence of stressors. There are multiple definitions of resilience, but resilience is defined in this study as “the ability, when faced with stress or adversity to actively use individual traits (internal factors) and wider social, community, and environmental supports (external factors) to return or maintain a positive state of mental health and functioning” [6]. However, some individuals may not recover to their original mental health state [7], whereas others may experience posttraumatic growth in the presence of the stressor [8].

Internal factors include personality characteristics and personal strengths, and external factors include the ability to rely on family, friends, school, and community [9]. Adolescents’ resilience increases when they use the combination of internal and external strengths to avoid negative outcomes in the presence of risks [6]. Previous models informed the conceptualization of resilience by Dray et al [6]. Lynch and Cicchetti [10] developed an ecological transactional model that supported a resilience framework. This framework emphasized specific levels of proximity in order from lowest to highest: society and culture, community settings, family environment, and the individual [10]. Lynch and Cicchetti [10] placed greater emphasis on internal resilience factors, but they acknowledged that the individual and environment constantly influence one another and could lead to changes within certain resilience domains. Gartland et al [11] built on the Lynch and Cicchetti [10] model and labeled these internal and external domains as separate from one another. Gartland et al [11] agreed with Lynch and Cicchetti [10] in that these factors could impact each other. Valuing the importance of individual and external factors, resilience is characterized as a dynamic, multidimensional concept that can change throughout one’s life span [11]. These internal and external factors have been targeted by resilience-enhancing programs [9,12].

Resilience-enhancing programs are mental health awareness programs that focus on social and emotional learning, mindfulness, and stress reduction [12]. Most of these school-based programs have shown positive intervention effects for depressive symptoms, internalizing problems, externalizing problems, and psychological distress [9]. Mental health promotion programs implemented at schools have demonstrated positive changes in resilience [12,13].

### Prior Work

Although universal resilience-enhancing programs are effective for adolescents, there would be more benefits for interventions that are easily accessible and specific to the individual user. Phone apps can serve as such interventions, as they are easy to use, can be tailored to individuals, and have shown positive effects for mental health outcomes. Furthermore, phone apps have demonstrated efficacy in positively impacting mental health, specifically adolescent anxiety and depression [14]. Most studies have focused on the feasibility of mental health apps, concluding that specific factors such as esthetics, ease of usability, and components of the apps are important in their adoption and use [14-16].

Regarding youth mental health apps, parents and other adults have reported concerns about safety and privacy [16-19]. Overall, 70% of the people who use mental health apps reported the importance of privacy and data encryption [20]. However, most mental health apps do not have any regulatory restrictions or address safety concerns [21]. Given these concerns, it is particularly important to include the consumers (adolescents) and stakeholders (parents, teachers, and clinicians) in the development and testing of these mental health apps [16].

Meta-analyses have provided evidence that phone-based mental health interventions that include self-monitoring components, are tailored to individuals, and increase social interaction are significantly more effective for adolescents than apps that do not use these features [15,22-24]. Self-monitoring involves the ability to log behaviors through the app [24]. Tailoring involves individualizing the experience for the user based on demographic information [22]. This differs from personalization, which is not as in-depth as tailoring in its approach to an individual’s characteristics [22]. Automatic dialogue components produced by avatars can increase social connection and have beneficial effects on outcomes [22]. However, although recent studies have identified features related to app efficacy, there are still no consensus guidelines for the core set of app features [25].

Youth apps have shown efficacy in reducing negative mental health outcomes [14]. The use of Ibobbly, an app aimed at reducing depression in adolescents and young adults [26], resulted in decreases in suicidal ideation, depression symptoms, and distress. Mobiletype [26] demonstrated an increase in the coordination of care between adolescent patients and pediatricians. At the time of this study, there were only 13 mental health apps whose use among adolescents was extensively studied (Table S1 in Multimedia Appendix 1 [23,26-37]), and none of them focused primarily on resilience. Out of these 13 apps, 5 (38%) focused on mental health treatment (eg, coping strategies for anxiety and decreasing suicidal ideation), 4 (31%) focused on self-assessment (eg, mood rating scales and tracking sleep quality), and 4 (31%) focused on mental health illness prevention (eg, prevention of anxiety disorders and prevention of psychosis). Among these 13 apps, 11 (85%) were in the formative and pilot stages, and 2 (15%) were specifically evaluated for mental health outcomes [26,27]. However, a meta-analysis stated that more mental health apps need to be tested among adolescents to determine their efficacy [14]. There is a need for a mental health app that incorporates these aspects and promotes the importance of resilience.

Adolescents and adults have identified barriers to using mental health apps in the past, so there is a need to gather input from these groups in designing a novel resilience-focused app. Conducting individual interviews and focus groups will assist in incorporating the app components that adolescents find helpful and will address some of the concerns related to using an app.

### Goals of This Study

This study aimed to develop and test the feasibility of a mental health and resilience-promotion app for youth. The study was conducted in 3 parts, with studies 1 (formative) and 2 (pilot) aimed at the development of the app and study 3 (evaluative) aimed at testing the app. The aims of studies 1 and 2 were to incorporate input from adolescents and stakeholders in the development of a feasible resilience app. Adolescents and stakeholders stated that the prototype was adequate and acceptable. This research adds to the literature by examining the core aspects that adolescents and stakeholders believe should be considered when developing a resilience app.

## Methods

### Recruitment

Study 1 was conducted from August 2019 to October 2019. Study 2 was conducted from November 2019 to January 2020. Study 3 was conducted from February 2020 to May 2020. For the participants who provided consent after the COVID-19 lockdown in March 2020, the consent form was discussed over the phone, and written consent was obtained electronically through a secure web-based platform. The participants were recruited through existing programs at an academic medical center using a combined convenience and purposeful sampling approach [38]. Given that adolescents were the target demographic, a more purposeful sampling approach was used to maximize demographic variety. Adult stakeholders were recruited primarily through convenience sampling. Inclusion criteria were as follows: adolescents who are aged 12 to 18 years, who can speak English, whose legal guardian can provide written consent, who can provide written informed assent, and who have a mobile phone. Exclusion criteria were as follows: children aged <12 years and adults aged >18 years, non–English-speaking adolescents, and adolescents without a mobile phone. Parental or guardian consent and participant assent were obtained before conducting any study procedures.

Inclusion criteria for parents, teachers, and clinicians were as follows: parents, teachers, and clinicians who can speak in English and have a mobile phone. Exclusion criteria were as follows: children aged <12 years and adults aged >18 years, non–English-speaking adolescents, and adolescents without a mobile phone. Some of the parents recruited were related to adolescents who participated in the study, which may limit the generalizability of the results. None of the interview content was shared with anyone outside the approved research staff per the institutional review board–approved protocol.

### Study Design

Studies 1 and 2 mainly used qualitative data. In study 1, interviews and focus groups were conducted to guide the development of preliminary content for prototype 1.0. Individual interviews can provide qualitative information about “why” people feel or answered in a certain way, which cannot always be captured through quantitative approaches [39]. Focus groups can be important as an adjunct to individual interviews because of the interactive component of having multiple people in the room and can generate new ideas that might not be captured within individual interviews [39]. The goal was to conduct at least 6 individual interviews to reach information saturation, as identified in previous research [40]. Overall, 5 interviews with adolescents, 3 interviews with parents, 3 interviews with teachers, and 3 interviews with clinicians were conducted. Each individual interview lasted 20 to 50 minutes. In addition, 1 focus group was led by 2 team members and included 6 adolescents. Previous qualitative studies encourage having at least 2 focus groups [41]; however, for pragmatic considerations, there was only 1 focus group per phase. The focus group interview lasted approximately 60 minutes. Individual interviews and focus groups followed a semistructured design. The interview guide was semistructured but also reflected key questions, themes, and concepts that the researchers determined a priori to be important for the development of prototype 1.0. To maximize interaction during focus groups, the team prepared primes and potential prompts to elicit response and interaction between participants, as well as anticipating techniques such as mirroring one participant’s comment when questioning subsequent participants. Study 1 interview questions are included in Multimedia Appendix 1.

In study 2, semistructured interviews and focus groups were conducted to ask the participants about the prototype. Given the iterative app development process, an updated prototype was developed during study 2. Prototype 1.0 was used for the individual interviews, and prototype 2.0 was used for the focus group. In total, 5 interviews with adolescents, 1 interview with a parent, 1 interview with a teacher, and 2 interviews with clinicians were conducted. Each individual interview lasted 25 to 50 minutes and involved the participant using the first version of the app (prototype 1.0) for at least 15 minutes on a tablet (iPad). One focus group was led by 2 team members and included 4 adolescents. Given the small size of this focus group, focus group coleaders called upon the participants to respond to questions to facilitate increased interaction and feedback about the prototype. The focus group interview lasted approximately 60 minutes, during which the group members were asked to explore an updated version of the app (prototype 2.0) on their phone for at least 15 minutes. Exploratory and confirmatory questions were used to help further refine the app before testing it for initial efficacy. Study 2 interview questions are included in Multimedia Appendix 1.

The participants were given US $25 for participation in the focus groups and US $50 for participation in the individual interviews. They completed 2 questionnaires (Computer System Usability Questionnaire [CSUQ] [42] and Mobile App Rating Scale [MARS] [43]), which obtained quantitative data about the participants’ thoughts surrounding app feasibility.

For study 3, our research team recruited 40 adolescents to use the app for 1 month. The adolescents completed questionnaires that measured their levels of resilience (Adolescent Resilience Questionnaire) [11], self-efficacy (Generalized Self-Efficacy Scale) [44], happiness (General Happiness Scale) [45], emotional awareness (Emotional Awareness Questionnaire) [46], coping skills (BRIEF COPE) [47], and perceived social support (Interpersonal Social Support) [48]. They were given weekly reminders via email to use the app with the hope of increasing their engagement, as shown to be effective within the Mobile Mood Diary [28]. They completed questionnaires before and within 3 days after using the app for 1 month. They were provided a compensation of US $50 for using the app and completing the prestudy and poststudy questionnaires.

### App

As referenced in the *Introduction* section, resilience-bolstering interventions increase the awareness about mental health concerns and focus on social and emotional learning, mindfulness, and stress reduction [12]. This app focused on enhancing resilience via 3 modules: depression prevention, stress management, and healthy lifestyle approaches. Each module follows a stepwise approach that includes the following questions: What is it? What does it look like? Where do I rate? How do I change it? and How can I practice? Each module includes videos from a teen and a clinician along with interactive games to learn skills within each section. The depression module teaches adolescents about the difference between sadness and depression. The module places emphasis on the symptoms that adolescents would experience if they were depressed. Adolescents can rate their depressive symptoms using a simple questionnaire (Patient Health Questionnaire-9) to obtain a total score regarding their depression severity (from none to severe). Adolescents can learn about specific strategies that can be helpful when they are depressed (eg, do an activity they enjoy) and can incorporate these strategies in the practice section (eg, do specific activity for certain number of minutes and rate mood before and after).

The stress module places emphasis on what stress consists of for an adolescent. The module provides psychoeducation about negative stress, positive stress, and recognizing when stress can lead to increased difficulties and anxiety. The module provides important information about the various ways in which stress presents in the body. Adolescents can rate anxiety symptoms with a simple questionnaire (Generalized Anxiety Disorder Scale-7) to obtain a total score regarding anxiety severity (from none to severe). Similar to the mood module, adolescents can learn ways to decrease their stress (eg, practicing relaxation) and can focus on this in the practice section (eg, taking a warm shower or bath).

The lifestyle module discusses the importance of self-care and the impact it can have on the quality of life. The module focuses on physical activity, nutrition, sleep, and mindfulness. Adolescents can rate themselves across these areas to see whether they are in a healthy range or if changes need to be made. Adolescents can learn about strategies to help improve their quality of life across these areas (eg, nutrition—think of food alternatives—turkey burger instead of regular burger) and can incorporate some of these changes in the practice section (eg, nutrition—listing times of mindful vs mindless eating).

### Ethics Approval

The University of Texas Southwestern Medical Center Institutional Review Board approved the study before the project commenced (STU2018-0304). Written informed consent and assent were obtained before any participant data were collected. All study participants had their protected health information deidentified, and the study data were only accessible by approved members on the research team.

### Data Analyses

A total of 4 independent raters and 1 qualitative expert were involved in transcribing and coding the audio recordings into the NVivo software (QSR International) [49]. The qualitative data were analyzed using a mixed applied thematic analysis [50]. An interview guide was created to reflect themes that had been referenced in previous mental health app research [16]. Kenny et al [16] identified multiple themes after targeted youth focus groups for their mental health app (CopeSmart). Their themes helped form our interview questions and initial coding list [16]. A deductive coding list was developed from the interview guide in consultation with a qualitative expert and the research team through a consensus discussion. Team members who served as independent raters first participated in a co-rating exercise for a set of 2 test set transcripts. Discrepancies among the team members about codes were highlighted and reviewed by the team to reach a consensus. After reviewing the test set of 2 transcripts, inductive codes were added (ie, social interaction and time) based on team member agreement. Each individual interview transcript and focus group transcript were reviewed by 2 independent raters. Once calibration had been achieved, 2 members of the study team coded each transcript and met to ensure consistency in coding. The NVivo (version 10.0) software was used to organize all coded material such that the material could be easily analyzed. This process was performed for both studies 1 and 2.

Our research team obtained means (and SDs) for feasibility through the CSUQ. Scores >4 provide evidence that the resilience app is acceptable. We also obtained the means (and SDs) for feasibility through the MARS. Scores >3 provide evidence that the resilience app is acceptable. Dependent samples 2-tailed *t* tests were conducted to determine whether there were any changes in resilience and secondary mental health outcomes among the adolescents before and after they used the app. P-P plots were constructed to test the assumption of normality for all the dependent 2-tailed *t* tests. Our research team obtained frequencies for the number of users who viewed the entire app, sections that the users viewed, and completion of specific modules.

## Results

### Overview

In study 1, we conducted 14 individual interviews (5 with adolescents, 3 with parents, 3 with teachers, and 3 with clinicians) along with 1 focus group (6 adolescents). In study 2, we conducted 9 individual interviews (5 with adolescents, 2 with clinicians, 1 with a parent, and 1 with a teacher) along with 1 focus group (4 adolescents). Additional demographic information is listed in Tables 1 and 2.

Information about the themes (with quotes) identified during studies 1 and 2 are presented in Tables 3 and 4.

**Table 1:** Study 1 demographics.

Variables			Values, n (%)
**Adolescent individual interview sample demographics (n=5)**			
	**Sex**		
		Female	3 (60)
		Male	2 (40)
	**Ethnicity**		
		Non-Hispanic	2 (40)
		Hispanic	3 (60)
	**Race**		
		White	2 (40)
		Asian	2 (40)
		American Indian	1 (20)
	**Grade**		
		7th grade	1 (20)
		8th grade	1 (20)
		9th grade	2 (40)
		10th grade	1 (20)
**Adolescent focus group sample demographics (n=6)**			
	**Sex**		
		Female	1 (17)
		Male	4 (67)
		Other	1 (17)
	**Ethnicity**		
		Non-Hispanic	4 (67)
		Hispanic	1 (17)
		Unknown	1 (17)
	**Race**		
		White	2 (33)
		African American	1 (17)
		Native Hawaiian	1 (17)
		>1 race	1 (17)
		Other	1 (17)
	**Grade**		
		7th grade	1 (17)
		9th grade	1 (17)
		10th grade	1 (17)
		11th grade	2 (33)
		12th grade	1 (17)
**Adult individual interview sample demographics (n=9)**			
	**Sex**		
		Female	8 (89)
		Male	1 (11)
	**Ethnicity**		
		Non-Hispanic	6 (67)
		Hispanic	3 (33)
	**Race**		
		White	7 (78)
		American Indian	1 (11)
		>1 race	1 (11)
	**Stakeholder**		
		Parent	3 (33)
		Teacher or school personnel	3 (33)
		Pediatric clinician	3 (33)

**Table 2:** Study 2 demographics.

Variables			Values, n (%)
**Adolescent individual interview sample demographics (n=5)**			
	**Sex**		
		Female	2 (40)
		Male	2 (40)
		Transgender	1 (20)
	**Ethnicity**		
		Non-Hispanic	5 (100)
	**Race**		
		White	3 (60)
		African American	1 (20)
		Asian	1 (20)
	**Grade**		
		8th grade	2 (40)
		12th grade	3 (60)
**Adolescent focus group sample demographics (n=4)**			
	**Sex**		
		Female	2 (50)
		Male	2 (50)
	**Ethnicity**		
		Non-Hispanic	4 (100)
	**Race**		
		White	2 (50)
		African American	1 (25)
		>1 race	1 (25)
	**Grade**		
		9th grade	1 (25)
		10th grade	2 (50)
		11th grade	1 (25)
**Adult individual interview sample demographics (n=4)**			
	**Sex**		
		Female	2 (50)
		Male	2 (50)
	**Ethnicity**		
		Non-Hispanic	4 (100)
	**Race**		
		White	3 (75)
		Asian	1 (25)
	**Stakeholder**		
		Parent	1 (25)
		Teacher or school personnel	1 (25)
		Pediatric clinician	2 (50)

**Table 3:** Themes and key quotes identified during study 1 interviews and focus group.

Theme	Adolescents	Adult stakeholders
Content	Having adolescents similar in age to talk about shared experiences, rather than having just videos Decreasing stress Importance of having teens discuss mental health experiences rather than having a generic narrator “I would expect the application to cover tips, ways to improve already...Having short quizzes to assess how I am doing and also for a way to track my progress would be helpful to see if I’m getting better with eating habits or how much sleep I am getting. Similar to most of the apps I use if I’m able to see I’m doing better than it encourages me to use the application more.” [Adolescent individual interview participant]	Difficulty brainstorming ideas in the absence of a prototype Importance of having adolescents learn self-care tips Navigate complex situations and use problem solving “Another component might be problem solving because I think in order to be more resilient, you need to have problem solving skills. For example, teaching simple acronyms on problem solving.” [Clinician interview participant]
Features	Less text and more images Easy navigation Strategy game with rewards Design and colors have to be appealing Have adolescent characters “Very eye-pleasing or eye-catching. The colors, adjust to what color you think would be better for you and being able to change the background to customize it.” [Adolescent focus group participant]	Stated importance of incorporating features that will keep youth excited Include adolescents as narrators “I think any videos or pictures just including people that look like [adolescents] would encourage them to use the app. So, whether it is race, body type, ability, gender, I think teens like when they can look at someone and say ‘Oh, that person looks like me.’” [Teacher interview participant]
Concerns	App should not require tracking multiple areas Stigma to use a mental health app “Something that would make me not want to use it is if it was asking me too many questions or asking too personal questions because that is just weird. Maybe you do have depression, but that does not give you the right to ask me questions the application does not need to know.” [Adolescent focus group participant]	Stigma from peers Cyberbullying through chat functions Promoting isolation “Having everything accessible via phone, you do not have to leave your home. It promotes isolation, which we know is not helpful for someone with depression or probably increases anxiety, so that is definitely a negative.” [Clinician interview participant]
Benefits	Help decrease stress Track changes in mood Help with anxiety surrounding school “If the [application] focused on stress and ways to relieve stress that would be very helpful for kids my age. Right now, is very stressful with huge tests and thinking about college. I think a lot of my friends would use it if had the [stress] aspect in it.” [Adolescent individual interview participant]	Wanted to focus on teen interview responses in terms of what would be beneficial
Most important thing	Adolescent individual interviews: make the app interactive Adolescent focus group: Appealing graphics “Make sure that the [application] is appealing to teens to come back. I have downloaded at least seven applications on my phone that talk about eating habits, exercising and I have probably opened them about two times now. If the [application] was able to draw me in especially with the progress thing, that would be the most important thing for me to keep going on the application.” [Adolescent individual interview participant]	Parents: appealing design to youth Teachers: have resources for youth Clinicians: make the app engaging “I would say focus on what the teens find interesting to them. What interests them and what would be the things they are looking for.” [Parent interview participant]

**Table 4:** Themes and key quotes identified during study 2 interviews and focus group.

Theme	Adolescents	Adult stakeholders
Content	Enjoyed the balance among videos, activities, and games Happy to learn about new coping skills when distressed Would not only use tips for themselves but would also share them with friends “I feel like I’ve had people tell me like very specific things that one of the two speakers mentioned, or I’ve like, they’ve told me about it and for instance, her friends did not know what to do. I also had no idea what to do or what to tell them. Just the list of I chose to listen to music or whatever, go for a walk, or listen to a happy song, all of those things. I was like okay, now I’ll have at least little suggestions for people when they ask or tell me issues like that.” [Adolescent individual interview participant]	Clinicians provided positive feedback about the rating scales Information was relevant for adolescents in their everyday life “Well, one I think it’s topical, pertinent. In school, working with these kids all day, there’s a need. So that is the first part. And the things that were presented in it. Kids need to hear it. I also liked, not only did it address depression and stress, but also talked about the lifestyle and how kids can look at what they’re doing now and what changes they can make. So, I liked that. It gave them options and was guiding, not just informative.” [Teacher interview participant]
Features	Logo Enjoyed color selection Concern text styles were too bold Lighter colors for the logo Main menu Good balance between colors and text Could have different images for the main menu icons Depression module game Most adolescents enjoyed the game Some were concerned that it was repetitive Wanted improvement with special effects	Logo Positive view of colors Worries about the font Replacing image with one that resembled resilience Main menu Easy to navigate and use Enjoyed same format in each of the modules Depression module game Enjoyed the game Concern that it was targeted for younger children Could have leaderboard to track scores
Concerns	Wanted easier access to surveys, activities, and games Minimal reasons for continued use No concerns about confidentiality “Because the application is very finite and once, I’ve done everything, I’ve done everything and it is done, so part of that having the everyday where it shows your progress over time. Even if you did not make the application bigger, it would expand that I would use this so much more because it feels like there is more to the application versus, I’ve watched all nine boxes that exist, and I’m done.” [Adolescent focus group participant]	Worried that adolescents would only use the app for a short period Concerned about the source of information Worried that the app cannot function as a solo intervention “I do not want the application to be the only thing a kid is seeking out if they are having issues with depression or stress. Maybe that is also something, like an activity that could be built into the application. Like to have them identify a trusted adult, like someone to go talk to and share with. I would not want a kid to solely rely on it.” [Teacher interview participant]
Engagement	Emphasized incorporating features that grab users’ attention Activities increased their interest in using the app “Well, animations usually make things more interesting for people to want to learn about something. And I know whenever we are watching videos in class, whenever it gets to an animated part, it is more interesting to watch.” [Adolescent individual interview participant]	Thought that the app was engaging One of the clinicians wanted more graphics within videos to maintain adolescents’ attention on the app “I liked that it was another form of interaction rather than just clicking and rating, which were the other types of interactions. It boosted my mood a little bit even though I was not in a bad mood so that was cool.” [Clinician interview participant]
Improvement	Wanted the option to connect to other users Interested in adding tabs to help users find engaging activities Potential inclusion of a journaling section “Maybe, if there was a way to connect to other teens. From personal experience, I rarely, if ever, recommend apps to somebody. But you could build a network and try to post. You could use teens and make them use their social media sites and share it, to give them a small incentive where they could share the application.” [Adolescent individual interview participant]	Wanted the inclusion of push notifications Thought that the app needed to be personalized “I think it could be personalized even more and that is the problem of where privacy and confidentiality come into play. You can personalize it more with open-ended questions or they can rate their mood. Have graphs to see how their mood is being tracked. There are apps themselves for tracking or websites. Personalize it more that way because right now educational is not as engaging for them to go back and review.” [Clinician interview participant]
Frequency of use	Said that they would use the app weekly Would log into the app after school or on the weekends Would use the app in specific situations Additions to the app would increase the frequency of use “I liked the application, but I feel like personally people would not use the app every day...I feel like there would definitely be some younger people that would. People who are just coming into anxiety would definitely use the application, but people who’ve had it for 5, 6 years would maybe not.” [Adolescent focus group participant participant]	Believed youth would use the app weekly Stated that adolescents would use the app after school or at night “Probably would not do it during school. Maybe after school, before bed. Probably not on the weekend so much if they are busy. Probably right before they go to bed or after dinner or after homework, maybe when they are done with school.” [Parent interview participant]
Recommendations	Most said that they would recommend the app to their peers One of the adolescent did not recommend the app The focus group participants were less willing to recommend the app “I think I would. I think it would be super helpful for a lot of people. Just trying to think of specific things. I like the idea of specific changes about the application, changes to make to your routine for life or someway. I would make that a bigger part of it. Yeah, because of the lessons talked about adding this to calendar or trying to incorporate different physical activities. I would make it a bigger part of the application, but other than that I would find it super helpful.” [Adolescent individual interview participant]	Clinicians discussed the benefits of the app with therapy One of the teachers viewed the app as an important addition youth could use with resources One of the parents did not see youth using the ap as currently constructed “Like I was talking about before, how approachable and easy to use it is. I would definitely recommend it because I like the idea of you know, saying things in session and having a way for them as a refresher and sometimes I feel like I have certain teens come in and they have so many things going on. Sometimes you have to skip some of the basic stuff and that is when I refer or recommend a book for them to read, but I would love to recommend an app. Where I could say go in and look at all the different symptoms and play around with it and see what is going on with you and how it relates to you.” [Clinician interview participant]

### Study 2 Quantitative Results

Changes from prototype 1.0 to prototype 2.0 included the removal of the “Rate Your Mood” function, changes in the depression module game (content and graphic updates), and improved smartphone adaptability. The participants rated the app as acceptable in the CSUQ (mean 6.30, SD 1.03) and MARS (mean 4.08, SD 0.61). Further analysis determined that the participants viewed the app as adequately engaging, functional, esthetically pleasing, informative, and impactful in the MARS. These results are presented in Table 5.

Exploratory analyses examined the participants’ ratings of the app and whether they would recommend the app to others. The participants gave the prototype a rating close to 4 stars after reviewing it for a short period (mean 3.85, SD 0.69). Of the 13 participants, 8 (62%) stated that they would recommend the app to “many people” or “everyone.” These findings are also presented in Table 5*.*

**Table 5:** Study 2 Computer System Usability Questionnaire and Mobile App Rating Scale scores (n=13).

Variables		Values
Computer System Usability Questionnaire (Likert scale from 1 to 7), mean (SD)		6.30 (1.03)
**Mobile App Rating Scale (Likert scale from 1 to 5), mean (SD)**		4.08 (0.61)
	App engagement	3.85 (0.67)
	App functionality	4.54 (0.60)
	App esthetics	4.28 (0.57)
	App information	3.90 (0.80)
	App impact on behavior	4.29 (0.59)
App rating (Likert scale from 1 to 5), mean (SD)		3.85 (0.69)
**Frequency of use over a year (Likert scale from 1 to 5), mean (SD)**		3.62 (0.96)
	1-2 times, n (%)	2 (15)
	3-10 times, n (%)	3 (23)
	10-50 times, n (%)	6 (46)
	>50 times, n (%)	2 (15)
**App recommendation (Likert scale from 1 to 5), mean (SD)**		3.84 (0.98)
	Few people, n (%)	1 (8)
	Several people, n (%)	4 (31)
	Many people, n (%)	4 (31)
	Everyone, n (%)	4 (31)

### Study 3 Results

A total of 40 participants provided consent for study 3 to use the app for 30 days. Overall, 60% (24/40) of the participants identified as female, 35% (14/40) as male, 2.5% (1/40) as transgender, and 2.5% (1/40) as nonbinary. Approximately half (19/40, 48%) of the participants identified as White, 20% (8/40) as Asian, 15% (6/40) as African American, and 5% (2/40) as American Indian. The nonusers (7/40, 18%), defined as the participants who never logged into the app, were removed from the analysis. Exploratory analyses revealed that there were no significant differences between nonusers and users in baseline resilience scores and demographic factors (eg, sex, race, and grade). An additional participant (1/40, 2.5%) who did not complete the poststudy questionnaires was removed from the analysis. The final analysis included 32 participants.

A total of 25% (10/40) of participants completed the entire intervention, and 55% (22/40) of participants completed some portions of the intervention. The depression module was completed by more users (18/40, 45%) than the stress module (13/40, 32%) and lifestyle module (10/40, 25%). As for each specific component, the “Where do I rate?” section was most used for the depression module (29/40, 72%), followed by the stress module (25/40, 62%) and lifestyle module (18/40, 45%). Adolescent use data are presented in Table 6.

The adolescents stated that the app was feasible overall in the CSUQ (mean 6.14, SD 0.84) and MARS (mean 3.94, SD 0.62). Further analysis determined that the adolescents viewed the app as adequately engaging, functional, esthetically pleasing, informative, and impactful in the MARS. The results are presented in Table 7.

No statistically significant improvements were found between the baseline and follow-up assessments in levels of resilience (mean 325.66, SD 59.35 vs mean 323.66, SD 60.30; *t*_31_=−0.96; *P*=.34) or levels of individual factors of resilience (mean 143.35, SD 28.94 vs mean 143.78, SD 27.58; *t*_31_=0.31; *P*=.76). The adolescents did not show statistically significant changes in any of the secondary mental health outcomes. Exploratory analyses provided evidence that the number of user log-ins was positively correlated with increases in individual resilience (*r*=0.39; *P*=.03).

**Table 6:** Study 2 adolescent use data (n=40).

Variable		Values
**App completion, n (%)**		
	None	7 (18)
	Some	23 (58)
	All	10 (25)
**Log-ins^a^**		
	Number of log-ins, average (SD)	3.83 (3.13)
	Number of log-ins among only users (n=33), average (SD)	4.64 (2.84)
	Number of log-in among users, range	0-14
**Module completion, n (%)**		
	Depression	18 (45)
	Stress	13 (32)
	Lifestyle	10 (25)
**Module use, n (%)**		
	Depression	29 (73)
	Stress	25 (63)
	Lifestyle	22 (55)
**Depression subsection completion, n (%)**		
	Introduction	20 (50)
	What is it?	20 (50)
	What does it look like?	19 (48)
	Where do I rate?	29 (73)
	How do I change it?	18 (45)
	How can I practice this?	19 (48)
**Stress subsection completion, n (%)**		
	What is it?	19 (48)
	What does it look like?	18 (45)
	Where do I rate?	25 (63)
	How do I change it?	16 (40)
	How can I practice this?	14 (35)
**Lifestyle subsection completion, n (%)**		
	What is it?	17 (43)
	What does it look like?	15 (38)
	Where do I rate?	18 (45)
	How do I change it?	10 (25)
	How can I practice this?	13 (33)

^a^Total number of log-ins: 153.

**Table 7:** Study 3 Computer System Usability Questionnaire and Mobile App Rating Scale scores (n=32).

Variables		Values
Computer System Usability Questionnaire (Likert scale from 1 to 7), mean (SD)		6.14 (0.84)
**Mobile App Rating Scale (Likert scale from 1 to 5), mean (SD)**		3.94 (0.62)
	App engagement	3.49 (0.82)
	App functionality	4.41 (0.65)
	App esthetics	4.17 (0.67)
	App information	3.90 (0.72)
	App impact on behavior	3.89 (0.90)
App rating (Likert scale from 1 to 5), mean (SD)		3.37 (0.83)
**Frequency of use over a year (Likert scale from 1 to 5), mean (SD)**		2.91 (0.96)
	None, n (%)	4 (13)
	1-2 times, n (%)	4 (13)
	3-10 times, n (%)	15 (47)
	10-50 times, n (%)	9 (28)
**App recommendation (Likert scale from 1 to 5), mean (SD)**		3.53 (1.24)
	Not at all, n (%)	2 (6)
	Few people, n (%)	4 (13)
	Several people, n (%)	11 (34)
	Many people, n (%)	5 (15)
	Everyone, n (%)	10 (31)

## Discussion

### Principal Findings

The aims of studies 1 and 2 were to incorporate qualitative and quantitative inputs from adolescents and stakeholders in the development of a feasible resilience app. When discussing the mental health apps, youth emphasized features over content. This finding is not surprising, given that previous apps with appealing esthetics have been viewed favorably by users [14-16]. It was also challenging for adolescents and adults to list ideas for content during study 1 interviews. For future studies, it may be important to provide general examples of content that could be included within the app to help generate further ideas from interviewees. The participants discussed improvements to facilitate user interest and engagement, which included logging features to track changes in mood. Turvey and Roberts [24] found that user interest in mental health apps increases with the option to track mood. Clinicians focused on improving engagement by incorporating interesting activities and a reward system. The lack of engagement has previously deterred youth from gaining the benefits of mental health apps [23,28].

The adolescents wanted the app to be personalized and suggested that there could be a “Get to Know You” section where users provide initial demographic information. Entering demographic and behavioral information has improved users’ app experiences [22]. The adolescents and clinicians discussed social connection as a vital feature to include within the app. Increased social interaction can lead to improved mental health app outcomes [24]. Morrison et al [22] stated that this can include increasing the variety of avatar automatic dialogue and mimicking peer-to-peer social interactions.

The users provided preferences for the resilience app content. The adolescents reported being happy that the prototype discussed relaxation skills. Youth mental health apps that teach coping skills have been viewed as more effective [27,29]. The adolescents enjoyed the surveys and discussed including components to track feelings through journaling. Previous mental health apps have emphasized the importance of self-monitoring components [22,24].

Although the prototype identified content strengths, the users were interested in specific changes. The interview participants inquired about including dilemmas to provide youth with opportunities to determine what they would do in complex situations. Wasserman et al [51] incorporated reviewing dilemmas within the Youth Aware of Mental Health program and found significant decreases in mental health symptoms.

The adolescents stated that an app would be beneficial if it could decrease their school stress. Youth stress from school has increased over the years and occurs from a multitude of interpersonal and academic pressures [52]. The participants expressed doubts about app use among youth because there was minimal incentive to continue using the app prototype. Any mental health app that is developed would benefit from including rewards, or adolescents may stop using it [53].

There was a consensus that multiple changes would make the app more appealing. With the ever-expanding world of technology, adolescents are more interested in apps with dynamic features. If we were able to incorporate all the feedback from study 2 to our study 3 prototype, we would have included the rate your mood function, improved navigation to engaging app functions, added youth dilemmas, provided app incentives, and added a variety of characters to increase social engagement.

The adolescents stated that the apps should include self-assessment and coping strategies. Morrison et al [22] and Whiteside [30] supported the notion that users enjoy immediate feedback from surveys. Fenwick-Smith et al [12] found initial evidence that adolescents benefit from programs that focus on enhancing their coping skills. Youth also reported interest in apps that facilitate social communication. Including automatic dialogue through avatars could increase adolescents’ connection with the app [22]. Mental health app developers should continue incorporating these areas in future apps.

Mental health app designers should be cautious when developing universal apps. Patwardhan et al [31] provided evidence that youth are more interested in mental health apps that can be tailored to their experience. Werner-Seidler et al [54] provided evidence that stepped-care approaches can be more personalized and helpful for youth. Even with universal apps, being able to tailor to the app to individuals can lead to beneficial user experience.

In general, apps should be used for resilience interventions to reach adolescents who may not receive this information elsewhere. In-person prevention programs have demonstrated efficacious changes for mental health outcomes [12,13,55]; however, many resources are needed for these programs. Youth mental health apps could be a target universal approach and decrease the burden on schools and primary care clinics, which have rigorous curriculum and health care demands.

The aims of study 3 were to investigate the feasibility of the mental health app and determine whether its use would lead to changes in resilience and secondary mental health outcomes. Although there were no statistically significant changes, the adolescents determined that the app was adequate, and use data provided important information about practical implications for future mental health app research.

The participants reported that the app was feasible through ratings in the CSUQ and MARS after 30 days of app use. These findings are a strength because the users found the app to be adequately engaging, functional, esthetically pleasing, informative, and helpful after prolonged use. This is an important first step, as apps must be rated as feasible before they can become effective [14-16].

There were no significant differences in resilience and other mental health outcomes after the adolescents used the app for 30 days. There are various reasons why there might have been no changes in user mental health outcomes. One of the possibilities is that the participants did not use the app enough, or the time given (ie, 30 days) was not enough to lead to mental health changes. Our exploratory analyses demonstrated a positive correlation between the number of times the users logged in and increases in internal resilience factors. Therefore, more app use could lead to changes in specific mental health outcomes. The users logged into the app once per week on average, but the researchers were unable to approximate the amount of time that the participants spent on the app. In addition, only 25% (10/40) of users completed all the app modules. In comparison, in the study by Tighe et al [27], 34 (56%) out of 61 users completed all the modules, which may be necessary for statistically significant mental health changes.

Specific features were missing from the app, which may have decreased app use. The app did not provide the participants the option to rate their mood or send them notifications. The researchers sent weekly email reminders to encourage the use of the app; however, the app was not designed to use SMS push notifications. Previous youth mental health apps have reported higher feasibility ratings and effectiveness when incorporating these components [23,28,31], so not having these features could decrease youth engagement in the app.

The adolescents appeared to prefer the depression module (18/40, 45%) over the stress (13/40, 33%) and lifestyle modules (10/40, 25%). One of the reasons for this is sample selection bias. This academic center had ongoing studies of depression in at-risk youth. Youth participants with a previous or current history of depression could account for the preference for the depression module. Adolescent depression prevalence is higher than adolescent anxiety rates [56], which could be the reason for the higher user interest.

Adolescents preferred the “Where do I rate?” section, which provided surveys that users could complete to receive feedback about possible depression symptoms, anxiety symptoms, and physical activities (eating, sleeping, and exercising). Whiteside [30] found that youth were more likely to complete self-assessments in comparison with practicing new coping skills. Surveys are more interactive and engaging to maintain youth’s attention, which is an important component for mental health apps [23,28].

### Limitations

Some limitations should be acknowledged within our sample. First, all the adolescent participants were recruited from an academic medical center setting. Recruitment from this setting may have created a sample selection bias, which could make findings less generalizable. Future app studies should focus on recruitment through schools and community centers to create diverse samples representing spectrum of mental health awareness. Although study 2 focus group participants were able to review prototype 2.0, our sample was small. More participants should have been recruited to make these findings for prototype 2.0 generalizable to adolescents. In addition, the users were only able to review the prototype for approximately 15 minutes.

Several limitations are the same for study 3. The researchers were unable to make significant changes from the prototype to the final version of the app; therefore, qualitative feedback from the users could not be fully incorporated. Study 3 participants did not participate in the interviews and focus groups after using the app for 30 days. Gaining qualitative feedback after study 3 was beyond the scope of the study, but these interviews would have provided more in-depth information about feasibility ratings and use rates for the app.

### Future Directions

Weekly use and nonsignificant changes in study outcomes provide support that this app could be paired in conjunction with an in-person component. School and primary care settings could have teachers and providers use the app videos to provide youth with psychoeducation about resilience and other mental health areas. In-person psychoeducation could be paired with asking youth to complete the interactive features of the app as homework within school settings. Pramana et al [32] received positive feedback from youth by pairing cognitive behavioral therapy and SmartCAT. The current version of the app may be more effective when combined with in-person interventions.

Exploratory analyses revealed a positive correlation between the number of times the users logged into the app and internal resilience changes. Increased use may lead to changes in resilience. Future mental health app developers could incorporate features and content that provide an interapp incentive to increase app use among youth. Pramana et al [32] suggested that goal setting with rewards can increase app use among youth.

The depression module was preferred among the users. Future mental health apps could incorporate psychoeducation and coping skills to alleviate depression, as adolescents may be more interested than stress or physical mental health factors. Tighe et al [27] reported that Ibobbly was effective in reducing depressive symptoms among older adolescents.

Study 3 participants preferred completing the “Where do I rate?” (eg, symptom surveys) section across the 3 modules. Whiteside [30] provided evidence that youth enjoy completing self-assessment in comparison with completing coping skill activities. Future mental health app developers could incorporate surveys and interactivity to increase user interest.

Future studies should obtain interview feedback from adolescents after they use the app for 30 days. Poststudy interviews could help researchers obtain insights into app features and content that impacted app use among adolescents. Although the app did not lead to changes in resilience and mental health outcomes, future studies should focus on the efficacy of pairing the content in additional settings. Apps have previously been viewed as efficacious in combination with mental health interventions [32,33]. Using this app with mental health treatment could lead to an increase in resilience.

### Conclusions

Studies 1 and 2 provided new information about feature and content development for a resilience app prototype. Practical implications for future mental health apps include incorporating adolescent feedback, making the app more engaging and personalized, and addressing any safety concerns from adolescent and adult stakeholders.

The adolescents found the resilience mental health app to be feasible after 30 days of use. Although there were no significant changes in mental health outcomes, the users logged into the app weekly and preferred the depression module and app surveys. These findings provide important practical implications to make future mental health apps more appealing and effective for adolescents.

This study is the first to test the feasibility and efficacy of a youth resilience app. New research has been conducted on specific features and content that adolescents would want to be incorporated into mental health apps. Although there were no significant changes in user mental health outcomes, the participants found the app to be functionally acceptable after using it for 30 days. The findings of this study provide researchers with important information for the development of youth mental health apps. Future studies should examine the efficacy of the app in conjunction with in-person mental health interventions.

## Appendix

Multimedia Appendix 1 Study 1 and study 2 interview questions.

## Abbreviations

CSUQ: Computer System Usability Questionnaire
MARS: Mobile App Rating Scale

## Data Availability

The data sets generated during or analyzed during this study are available from the corresponding author upon request.
