# Supplementary material for: Examining a Resilience Mental Health App in Adolescents: Acceptability and Feasibility Study
Source: JMIR Form Res. 2023 Mar 22;7:e38042. doi: 10.2196/38042 (PMC10132019; doi:10.2196/38042)
Supplement: Multimedia Appendix 1 [file formative_v7i1e38042_app1.docx]

**Multimedia Appendix**

This is a Multimedia Appendix to a full manuscript published in the J Med Internet Res. For full copyright and citation information see http://dx.doi.org/10.2196/jmir.38042

**Table S1.** List of the 13 Mental Health Apps.

| Reference | Name of the app | App aims and objectives | Research design | Findings |
| --- | --- | --- | --- | --- |
| Kenny et al [23] | CopeSmart | - Foster positive mental health through (1) emotional self-monitoring and (2) the promotion of positive coping strategies - Contains multiple sections, including Rate My Mood, Coping Tips, Resources, and Mood History | - 43 participants in Ireland (88% female), aged 15-17 years, gathered information about feasibility and acceptability | - 79% of the participants liked the app; 93% said that it was easy to use; and 70% would use the app in the future - Participants used the app on 4 of 7 days - Evening (6-9 PM) was the most popular time for using the app - Rate My Mood was the most useful part of the app - Some participants found Coping Tools and Resources to not be helpful (23% and 40%) |
| Kennard et al [34] | Safety Plan App | - Develop a brief inpatient intervention for suicidal adolescents | - Gathered qualitative data from 5 teenagers and clinicians to focus on the benefits of and concerns about the Safety Plan App for teenagers | - Clinicians said that they were comfortable with patients having safety information on their smartphone - Worried about privacy or confidential terms - Parents thought that a smartphone app for safety planning would be convenient and easily accessible and would improve safety - Teens reported that they would be comfortable using an app - All the participants endorsed a phone app for improving the accessibility and portability of the patient’s safety plan |
| Tighe et al [27] | Ibobbly | - In module 1, the participants learn to identify thoughts, feelings, and behaviors - In module 2, the participants were taught to regulate their emotions through mindfulness, acceptance, and self-soothing activities - In module 3, the participants were aided in identifying values important to them and asked to set small, achievable goals to help them live their lives | - RCT^a^ for the efficacy of the mental health app | - Significant preintervention and postintervention differences in the treatment arm for SI^b^ - The participants in the Ibobbly arm showed a substantial and statistically significant reduction in PHQ-9^c^ scores compared with the waitlist controls - Significant change in general psychological distress in the Ibobbly arm - Of the 40 participants whose usage data were available, 34 completed all the modules and assessments - Significant reductions in depression and psychological distress but not in suicidality or impulsivity |
| Reid et al [26] | Mobiletype | - Mobile health assessment and management app that monitors mood, stress, and everyday activities and transmits the gathered information to PCPs^d^ through a secure website | - RCT for the efficacy of the mental health app | - When the participants completed the program, PCPs gained a better understanding of patients’ functioning - Compared with attention comparison group, app provided help with medication choices, referrals, diagnosis, and mental/health problems - Findings suggest that a mobile phone monitoring program that captures and summarizes detailed and specific mental health information and more general health information in a time-efficient manner may assist PCPs in the management of youth mental health problems - Young people thought that it was beneficial to send info to PCPs |
| Løventoft et al [35] | Daybuilder | - Smartphone intended to support people with depression by monitoring their daily lives and supporting their interactions with the community - It included mood tracking along with lifestyle factors, life events, and medication management | - 6 participants aged 17 to 24 years completed the study and provided information about the feasibility and acceptability of the app | - Information about attitude toward the prototype was collected - Individuals liked the idea of an app in the style of Daybuilder but had reservations about some aspects of the app and usability and software concerns. |
| Matthews and Doherty [28] | Mobile Mood Diary | - The participants could rate their mood, energy, and quality of sleep and were sent SMS reminders to do so - Diary could involve text entries as well | - Focus groups- about the prototype with children aged 12 to 14 years | - Major usability issues were found - In the second portion of the study, 21 kids from inner-city schools used the app - The app group had higher adherence than the paper group - The app was easy to use, and the participants had minimal worries about privacy - Clinical evaluation of MMD^e^ with therapists and clients: 9 participants over span of 2 years; higher adherence than paper versions of diary; found it to be “engaging” - Therapist wanted a buddy system owing to a lack of experience with using technology in treatment - Enjoyed graphs of what their mood looked like over the past 2 years - Teens expressed concern over using the app owing to disclosure of name - Provide options for use (paper, mobile, and desktop) |
| O’Brien et al [29] | Crisis Care | - Crisis Care has an adolescent mode and a parent mode designed to be used in tandem when the adolescent is experiencing a suicidal crisis following discharge from an ED^f^ or inpatient psychiatric unit - Adolescent mode gave immediate access to a set of coping skills identified as being helpful during a suicidal crisis. The “My Skills” section included Call an Adult, Relax, and Laugh and Do Something - Adolescents were asked to use the “Help Me Now” section if they felt at imminent risk of self-harm - Parent Mode of Crisis Care gives access to tips on how to effectively listen to suicidal adolescents, helps parents coach adolescents in their personalized coping skills, and gives immediate access to professional help and consultation | - 20 parent-adolescent dyads participated in this study for feasibility and acceptability | - High ratings of feasibility - Individuals found the app to be useful during a crisis and were satisfied with the content - Changes were made to the app to improve ratings |
| Patwardhan et al [31] | REACH | - REACH for Personal and Academic Success is an indicated prevention and early intervention program targeting anxiety disorders and related problems in youths - Activities included daily diary | - 22 youths from public schools participated in the “system usefulness, satisfaction, and ease” aspect of this research - Reach app was highly and positively rated - REACH is capable of deploying notifications relevant to skill practice and offers tools for personalizing and tailoring the protocol - Higher ratings for the quality of support information, system ease of learning, and system satisfaction | - REACH app was highly and positively rated - REACH is capable of deploying notifications relevant to skill practice, offers tools for personalizing and tailoring the protocol, and has higher ratings for quality of support information, system ease of learning, and system satisfaction |
| Whitehouse et al [36] | TickiT | - Develop an app that assessed adolescents’ current psychosocial levels through screening questions that could be sent to PCPs or other providers | - 80 adolescents aged 12-18 years helped with the cocreation of screener along with gathering information about usability and feasibility | - Talked to health care providers as well as IT administrators - Teens wanted colorful interface that was “different from school” and gender neutral colors - 92% of the adolescents said that the screener was easy to use, found question easy to understand, and were comfortable answering questions - Residents felt positively about the instrument, and surgeons did not like the measure |
| Pramana et al [32] | SmartCAT | - Smartphone app that cues youths to use the CBT^g^ skills taught in sessions - Web-based portal that allows therapists to monitor skill use and send cues and treatment-related materials to engage youths in real time via secure messages - Communication protocol that allows real-time bidirectional exchange between the app and the portal - App incorporates authentication and encryption for security purposes - Use of reward bank | - Pilot study with 9 youths (aged 9 to 14 years) - Initial information about adherence to the app was gathered | - Data showed that the app was used frequently during treatment - Patients were compliant with brief CBT protocol - App was rated as highly usable - Goal setting through rewards can incentivize kids to use the app |
| Whiteside [30] | Mayo Clinic Anxiety Coach | - Self-help app designed to meet individuals’ needs through exposure-based CBT - Self-evaluation module measures the frequency of anxiety symptoms with a self-report Likert-type scale - Second module contains psychoeducational material - Third module focuses on exposure exercises | - 169 children and adolescents aged between 5 and 17 years downloaded the app in the past year | - 70% used the app between 4 and 20 times - Individuals were more likely to take the self-assessment than to create a fear ladder or complete an exposure |
| Hides et al [37] | Music eScape | - App designed to help people identify, express, and regulate their emotions - Users take music from their own song libraries to create playlists that reflect level of valence (pleasant to unpleasant) and arousal (very low to very high) | - 169 adolescents and young adults (aged 16 to 25 years) used the app - 84% of these participants completed follow-up data at 6 months | - Users said that the app had high objective level; good engagement, esthetics, information; and acceptable functionality - There were improvements in emotion regulation strategies, mental distress, and well-being over the 6 months |
| Traber-Walker et al [33] | Robin | - App used in conjunction with a standardized manual to target adolescents who were at risk for developing psychosis | - A prototype of the app was tested with patients (N=7, aged 14-18 years) and clinicians treating early psychosis | - Patients reported interested in using the app daily - Clinicians wanted to incorporate the app within therapy - Modules containing information about symptoms and coping strategies were the most used |

^a^RCT: randomized controlled trial.

^b^SI: Suicidal Ideation

^c^PHQ-9: Patient Health Questionnaire-9.

^d^PCP: Primary Care Physician

^e^MMD: Mobile Mood Diary

^f^ED: Emergency Department

^g^CBT: cognitive behavioral therapy.

*Study 1 Interview Questions*

1. Intro to interview: “Apps have become an important part of everyday life because they seem to be fun, enjoyable, and easy to use.” What has your experience been like using phone apps? (Apps)
2. What parts of phone apps do you find appealing or enjoy the most? (Apps)
3. What app do you enjoy using the most? (Apps)
   1. Follow-up Question: Why do you like using that app?
4. (Transition topics) “So we’ve talked about general apps, but I want to switch gears for these next questions.” Would you be interested in using a mental health phone application? (Mental Health Apps)
   1. Follow-up Questions: What would make you interested?
   2. What would be helpful or (not helpful) about it?
   3. Do you think other teens like you would feel the same way about a mobile app?
   4. For instance, would you use it during the day? Would you feel comfortable using it, for example, at school if you needed to?
5. What would be an incentive for you to using a mental health application? (Usability and Acceptability)
6. How would you stay engaged when using a mental health application? (Usability and Acceptability)
7. What types of graphics and visuals would you find helpful when using a mental health application (e.g., interactive text, video, pictures, combination, provide examples if needed)? (Usability and Acceptability)
8. What components/aspects would make it easy for you to use a mental health application? (Usability and Acceptability)
9. What are your potential concerns about using a mental health application? (Safety and Privacy)
10. Would you be uncomfortable about using a mental health application? (Safety and Privacy)

a) Follow-up Statement: Tell me more about that

1. Introduction of resilience application: “Some apps are fun to use; some apps make your life easier. Now that we’ve talked about that, let me introduce to you to the resilience application. The application will have 3 sections that will focus on depression, stress, and lifestyle factors (i.e., nutrition, exercise, sleeping) and will have teens/adults providing information but also involve activities that help with resilience.”
2. What does resilience mean to you (if you know)? (Resilience)
   1. Provide definition of resilience if they do not know: Resilience means the ability to “bounce back” when something bad happens
3. If a phone application was focused on improving resilience, what would you expect it to cover? (Resilience)
4. What is the single most important thing designers should focus on

when making this phone application for resilience? (Mental Health App Design)

*Study 2 Interview Questions*

1. What are your initial thoughts after looking at this application for a short period of time? (General Feedback)
2. What did you like about the application? (General Feedback)
3. What did you dislike about the application? (General Feedback)
4. Do you have any concerns when using this application? (App Concerns)

a. Follow-up Question: What are some of those concerns?

Show Main Icon (Print this out)

1. Tell me what you think about this image/icon.
2. What kind of image would be best for the icon?
3. Would you suggest something else?

We’d want a teen to be able to find the icon easily, but would that make it too obvious… this is also why we asked you about confidentiality. Does this change what you think about an image/icon?

Show Main Menu (Print out screenshot)

1. What were your thoughts about the main menu?
2. What would you suggest to add / change?
3. Are there strategies that you use that are not on this menu?

Show Sub Menus (Print out screenshot)

1. Do you think these strategies would be helpful to you?
2. What would you suggest to add / change?
3. Are there strategies that you use that are not on this menu?
4. What do you think about the design and graphics? What about the game?

Show picture of teen actor (Print out screenshot)

1. Did you like the teen actor? Why or why not?
2. Is she relatable? Why or why not?

Show picture of doctor (Print out screenshot)

17) Did you like the doctor? Why or why not?

18) Is she relatable? Why or why not?

Usability:

1. Would you feel comfortable using this app? Why or why not?
2. When would you use it?
3. What would help you to make this part of your daily life for the period of time you and your doctor think you need it?
4. Would you recommend this application to teens? (App Recommendation)

a. Follow-up Question: Why would you choose to recommend or not recommended the application to others?
